# Asthma, Food Allergy, and How They Relate to Each Other

**DOI:** 10.3389/fped.2017.00089

**Published:** 2017-05-09

**Authors:** Ru-Xin Foong, George du Toit, Adam T. Fox

**Affiliations:** ^1^Division of Asthma, Allergy and Lung Biology, Department of Paediatric Allergy, King’s College London, Guy’s and St. Thomas’ Hospitals NHS Foundation Trust, London, UK; ^2^Institute of Child Health, University College of London, London, UK

**Keywords:** asthma, food allergy, children, wheeze, anaphylaxis

## Abstract

The association between atopic diseases is well known, and previous research has shown that having one atopic disease can predispose to having another. The link between asthma and food allergy has been well researched, but the exact relationship between the two atopic conditions is not fully understood. Food allergic infants are at increased risk for the development of asthma and are at risk of food-induced asthmatic episodes and also anaphylaxis. Having a diagnosis of both food allergy and asthma has also been shown to have an effect on the severity of a patient’s disease including being at greater risk of severe asthmatic episodes. Therefore, understanding the relationship between these two conditions in order to treat and manage these children safely is crucial to clinicians.

## Background

Diseases including asthma, eczema, allergic rhinitis, and food allergy are typically considered as allergic diseases, although the exact association with atopy is frequently debated for eczema and asthma. Nonetheless, such diseases commonly coexist and are common in pediatric populations worldwide. Children affected with one allergic disease frequently develop other allergic diseases. The sequence of disease progression is often referred to as the “atopic march” (1). For example, infants with eczema are at higher risk of developing food allergy, children with egg allergy are at increased risk of developing allergic respiratory diseases, and children with allergic rhinoconjunctivitis are at increased risk of developing asthma. Furthermore, children with a single food allergy frequently develop additional food allergies. The causal relationship between these atopic diseases remains unclear as it is not absolute in all patients and the sequence may vary (2–5). The dual-allergen hypothesis provides a plausible explanation as to how allergic disease may progress. It describes how early allergic sensitization occurs through breakdown of skin barrier integrity that allows for exposure to food and environmental allergen, an effect that can be moderated be early-life ingestion for foods such as peanut and is some studies hens egg (6–10). Research has shown that the development of eczema can be associated with mutations of the filaggrin gene that is responsible for a major structural protein in the epidermis (11). Thus, children with eczema are at greater risk of developing food allergies due to a weakened skin barrier; for example, a study reported that 50% of children with eczema developed food allergy by 1 year (12). Similarly, Martin et al. (13) showed that in infants with eczema, the earlier onset and greater severity of their eczema symptoms increased their risk of food allergy. This emphasizes the hypothesis that allergen exposure through the cutaneous route contributes to allergic sensitization and highlights the key role of skin barrier integrity in protecting the infant immune system, which has been seen in children sensitized to peanut allergy (14, 15). A review showed that approximately 70% of patients with severe eczema developed asthma or allergic rhinitis later in life, and asthmatic patients who had filaggrin mutations had a difficult disease course with more asthma exacerbations (11). Other work has shown that patients who have filaggrin loss-of-function mutations have a significant association with food challenge confirmed peanut allergy (16).

### Asthma

Asthma is one of the most common long-term childhood conditions of which approximately 9% of children are affected by it (17). Asthma is defined as a chronic respiratory disease characterized by recurrent attacks of wheeze and breathlessness. These symptoms occur due to irritation that occurs in the airways causing inflammation and swelling resulting in reduced lung airflow (18). Over time with advances in asthma medicine, management continues to change but treatment primarily focuses on assessment of asthma severity, the use of acute and chronic medications including bronchodilators, anti-inflammatory medication (i.e., steroids), and treatment of comorbidities (19). In the management of acute asthma, the goals are to reverse airflow obstruction, correct significant hypoxia, and prevent future relapses (20). In order to achieve this, management for acute exacerbations includes the use of oxygen, short-acting inhaled beta-agonists, ipratropium bromide, systemic corticosteroids, and magnesium sulfate. With regards to long-term management of asthma, stepwise escalation strategies following regular symptom assessment and lung function tests include using medications such as inhaled long-acting beta-agonists, inhaled and systemic corticosteroids, and leukotriene-receptor antagonists (21).

### Food Allergy

In the last three decades, there has been a worldwide increase in the prevalence of food allergy with 3.5–8% of children having food allergies (17, 22, 23). Food allergy is defined as an adverse immunological reaction that occurs on exposure to a food that re-occurs on repeat exposure (22). It is usually classified into immunoglobulin-E (IgE)-mediated food allergy, non-IgE-mediated food allergy, or mixed IgE- and non-IgE-mediated allergy. IgE-mediated allergy has an acute onset (within 2 h of exposure), and presenting symptoms are often respiratory, skin, and gastrointestinal in nature, whereas non-IgE-mediated food allergy has a delayed onset of symptoms (from 1 to 24 h), and the symptoms tend to be skin and/or gastrointestinal (24). The key investigations for diagnosing food allergy include taking a thorough clinical history, skin prick testing, serum-specific IgE, and the double-blinded oral food challenge, which considered the gold standard investigation (25). For food allergies proven by positive oral food challenges, recommendation is for strict avoidance of the causative allergen. For those patients who experience life-threatening symptoms of IgE-mediated food allergy, an emergency self-injectable adrenaline device is often prescribed as well. However, over the last 10 years, increasing research has been performed looking at the use of oral, sublingual, and epicutaneous immunotherapies to desensitize patients through tolerance induction (26).

### Understanding the Relationship between Food Allergy and Asthma

Asthma and food allergy have been commonly shown to coexist with each other, especially as they often share risk factors (family history of allergy, atopic eczema, and asthma) but the way in which they interact and influence each other is yet to be fully understood. Studies have shown that food allergies can develop in the first year of life and precede the development of asthma (17, 27). There has also been increasing recognition that there is an allergic component to asthma as a disease with particular focus on the role of environmental allergens (i.e., house dust mite and cat allergens). Exacerbations of asthma can be caused by exposure to inhalant allergens, although avoidance of these allergens on asthma disease is not entirely understood. For example, a study that looked at the effectiveness of avoiding house dust mite allergen on asthma management found that using allergen-impermeable covers to avoid house dust mite allergen did not have a significant effect on clinical asthma (28). Schroeder et al. showed that there was a higher prevalence of asthma in children with food allergy as well as it occurring at a 7 earlier age compared to children without food allergy (29). Another study showed that compared to children who were not sensitized to common food and aeroallergens, those who were cosensitized had a higher risk of developing respiratory allergic disease (27). Studies have also looked at the timing of when food sensitization occurs and have shown that food sensitization early in life (within the first 2 years of life) is a strong predictor of allergy by school age and also children with food allergy have approximately double the chance of developing asthma and rhinitis (30, 31).

There also seems to be an association between asthma and non-IgE-mediated food allergy, although it is less prevalent than that seen in IgE-mediated allergy (32). In a study, approximately one-third of children with non-IgE-mediated food allergy had asthma and allergic rhinitis (33). Higher rates of asthma (26–66%) have also been reported in eosinophilic esophagitis, which is considered a food allergy disease (34–36).

## Asthma Triggered by Food Allergens

The way in which food allergens may trigger asthma symptoms is not fully understood. The respiratory symptoms that occur in food allergic reactions commonly include rhinitis, bronchospasm, cough, and laryngeal edema (17). One theory is that particles of ingested food are inhaled into the airway, and exposure of these allergenic proteins to mast cells in the lungs causes inflammation and therefore respiratory symptoms (17, 24). Commonly documented respiratory reactions from aerosolized food proteins have been well documented over the years, mostly in adults. One of the most commonly described examples is baker’s asthma where exposure to inhaled flour proteins causes an IgE-mediated type reaction, which manifests as asthmatic symptoms. Diagnosis is based on a history of work-related asthma symptoms, skin prick tests, and inhalation challenges to bakery allergens, which is the gold standard test (37). Aerosolized fish protein allergens have also been detected in open-air fish markets and can cause respiratory related symptoms due to inhalation of fish proteins (38). Similarly, occupational asthma and allergy has been reported in snow crab-processing workers who on cumulative exposure to snow crab have developed symptoms of asthma and allergy (39). With regards to children, Roberts et al. performed bronchial challenges in children with proven IgE-mediated food allergy and asthma using aerosolized foods (40). In this study, despite dietary avoidance of allergens (i.e., fish, milk, eggs, chickpeas, and buckwheat), the children had worse chronic asthma symptoms when there was environmental exposure to the foods (i.e., families cooked with the allergenic foods at home). However, when the families stopped cooking the allergenic food(s) at home, the child’s symptoms improved, and they needed less inhaled corticosteroid treatment (40).

There has also been research performed on specific food allergens and their association with respiratory symptoms and the development of asthma. For example, in a large birth cohort study, having egg allergy during infancy was predictive of respiratory allergy later in childhood (2). In fact, they reported a positive predictive value of 80% if the child also had eczema (2). Rhodes et al. found that in a study of 100 infants who were deemed at high risk of developing asthma and atopy (i.e., had atopic parents), those who were sensitive to egg and milk in the first year of life was predictive of having asthma as an adult (5). Another study looking at peanut or tree nut allergies showed that patients with a severe history of asthma were at greater risk of life-threatening bronchospasm occurring after ingestion of nuts (*p* < 0.0001) (41).

### Factors Contributing to the Development of Food Allergy and Asthma

In children, 4–8% of asthmatic patients have food allergies and approximately 50% of those with food allergies have allergic reactions that involve acute respiratory symptoms (24, 42). Various factors have been found to affect the risk of developing asthma in children with food allergy but some of the key factors include seasonal changes, the host immune response, and the use of anti-IgE treatments. Asthma exacerbations seem to coincide with seasonal changes, for example, increase aeroallergen levels such as grass pollen in the spring (43) and dust mite in the autumn (44). The specific-IgE antigens bind to mast cells and basophils causing an inflammatory response within the airways, which over time can cause airway modeling (45). The host immune response to allergens activates an inflammatory process causing allergic cytokines to be released and a subsequent rise in IgE levels, which have been shown to be associated with an increased risk of asthma (45, 46). In one study, they found that high IgE levels at 6 months old was associated with early-onset of asthma and also a strong relationship with the development of asthma in school-age years (47, 48). Milgrom et al. showed that 90% of children in their study with asthma had positive skin prick tests to common allergens (45). Also, the use of anti-IgE treatment (i.e., monoclonal anti-IgE antibodies such as omalizumab) in asthmatic patients has been successful, which suggests there is a role of IgE in the pathogenesis of asthma. There have been studies that have shown that omalizumab is effective in reducing the need for corticosteroids including complete withdrawal of corticosteroids compared to placebo, a reduction in asthma-related symptoms, and improvement in quality of life of these patients (45, 49).

### Morbidity and Mortality in Asthma

In adults, there is evidence that being allergic to more than one food is associated with an increase frequency of oral steroid use and a higher risk of lifetime hospitalizations and emergency department attendances (50). In children, a study showed that having a milk or peanut allergy was associated with an increased number of hospitalizations (*p* = 0.009, 0.016) (51). More specifically, having a milk allergy was associated with an increased use of systemic steroids (*p* = 0.001) (51). Simpson et al. also showed that children with asthma who also had peanut allergy had a 1.59 times greater rate of systemic steroid use and 2.32 times greater rate of hospitalization (52).

There is also evidence that suggests that exposure to food allergens can be a risk factor for life-threatening asthma. For example, in a study of children with peanut allergy, 9% (4/46) of the children died from an exacerbation of asthma that represents a significantly higher fatality rate for an asthmatic population (53). Roberts et al. compared children aged 1–16 years with life-threatening asthma (defined as requiring admission to pediatric intensive care) to those without non-life-threatening asthma and showed that life-threatening asthma was significantly associated with having food allergy (OR 5.89, 1.06–32.61) and having multiple previous admissions for asthma (OR 9.85, 1.04–93.27) (54). Ernst et al. conducted a study in patients aged 5–54 years and 129 of the patients had “fatal” asthma. The main finding in this study was that over 10 prescriptions or more of bronchodilators was associated with an increased risk of near-fatal asthma, but they also found that food allergy was an independent risk factor for near-fatal asthma (odds ratio 5.1, 95% CI 2.4–11.1) (55). Similarly, a case–control study showed that patients with near-fatal asthma (defined as requiring ventilation on intensive care unit) were more likely to be food allergic (OR 3.6, 1.6–8.2) and/or have had anaphylaxis (OR 5.6, 2.7–10.6) (56). Vogel et al. compared children who had ward-based care or ambulatory care (i.e., no hospitalization required) with children with potentially fatal asthma (requiring pediatric intensive care admission) and also found food allergy to be a risk factor for life-threatening asthma (57).

Asthma has also been identified as a risk factor for anaphylaxis and is associated with poorer outcomes in children with food allergy (24). Boyano-Martínez et al. conducted a study where children with cow’s milk allergy had a 10 times higher chance of a severe reaction if they also had asthma (58). Another study found that the majority of fatal reactions attributed to food allergy were asthmatic reactions that occurred in patients on daily asthma treatment (59). Furthermore, in a series looking at fatalities due to food-induced anaphylactic reactions, the majority of the children were asthmatic, and their respiratory symptoms were identified as the main cause for the severity of their reactions (60). With this evidence over time, guidelines have been produced by various organizations of which pediatric allergists are recommended to prescribe self-injectable adrenaline devices to patients who have both food allergy and asthma (24, 61, 62).

There have also been studies looking at preventative measures [i.e., house dust mite avoidance measures (i.e., protective mattress covers), allergen food avoidance] to prevent atopy in children, of which there is some evidence showing a reduction in respiratory symptoms (i.e., nocturnal cough, severe wheeze) by 1–2 years of age (63–65). Some studies suggest that immunotherapy to respiratory allergens can prevent allergic sensitization to new allergens, but this has not been observed in all studies (66, 67).

### Clinical Implications for Patients

When reviewing food allergic or asthmatic children, a detailed clinical history should be taken to identify potential triggers for both allergic disease and asthma. If a specific trigger can be identified, primary advice is for avoidance of the allergen. Asthmatic patients with food allergies require regular assessments, careful monitoring and dietary and emergency management plans, review of treatment, and medication adherence reviews. In cases of status asthmaticus, the use of intramuscular adrenaline should be considered if there is a history of food allergies. Equally, patients who have known food allergies with respiratory symptoms should be offered beta-agonist inhalers.

## Conclusion

Children who have both food allergies and asthma are at an increased risk of severe asthmatic episodes and may be at greater risk of food-induced anaphylaxis and also food allergen-triggered asthmatic episodes. It is important that clinicians educate patients appropriately regarding the higher risk of life-threatening asthma and anaphylaxis and ensure they receive regular assessments regarding their treatment and management.

**Summary of key points**

- Food allergic infants are at increased risk of developing asthma.
- Children with both asthma and food allergies are at increased risk of severe asthmatic episodes.
- Children with both asthma and food allergies may be at greater risk of allergen-triggered asthma episodes and food-induced anaphylaxis.
- Patients with known IgE-mediated food allergies and asthma should have immediate access to self-injectable adrenaline and inhaled beta-agonists.

## Author Contributions

All the authors (RXF, GdT, and AF) were involved in the writing and editing of the manuscript.

## Conflict of Interest Statement

The authors declare no conflicts of interest regarding the writing of this mini-review.
